# Phylostratic Shift of Whole-Genome Duplications in Normal Mammalian Tissues towards Unicellularity Is Driven by Developmental Bivalent Genes and Reveals a Link to Cancer

**DOI:** 10.3390/ijms21228759

**Published:** 2020-11-19

**Authors:** Olga V. Anatskaya, Alexander E. Vinogradov, Ninel M. Vainshelbaum, Alessandro Giuliani, Jekaterina Erenpreisa

**Affiliations:** 1 Department of Bioinformatics and Functional Genomics, Institute of Cytology, Russian Academy of sciences, 194064 St. Petersburg, Russia; 2 Department of Oncology, Latvian Biomedical Research and Study Centre, Cancer Research Division, LV-1067 Riga, Latvia; ninela.vainselbauma@biomed.lu.lv; 3 Faculty of Biology, University of Latvia, LV-1586 Riga, Latvia; 4 Istituto Superiore di Sanità, 00161 Rome, Italy; alessandro.giuliani@iss.it

**Keywords:** polyploidy, unicellularity, early multicellularity, embryonality, cancer, bivalent genes, viral-origin oncogenes

## Abstract

Tumours were recently revealed to undergo a phylostratic and phenotypic shift to unicellularity. As well, aggressive tumours are characterized by an increased proportion of polyploid cells. In order to investigate a possible shared causation of these two features, we performed a comparative phylostratigraphic analysis of ploidy-related genes, obtained from transcriptomic data for polyploid and diploid human and mouse tissues using pairwise cross-species transcriptome comparison and principal component analysis. Our results indicate that polyploidy shifts the evolutionary age balance of the expressed genes from the late metazoan phylostrata towards the upregulation of unicellular and early metazoan phylostrata. The up-regulation of unicellular metabolic and drug-resistance pathways and the downregulation of pathways related to circadian clock were identified. This evolutionary shift was associated with the enrichment of ploidy with bivalent genes (*p* < 10^−16^). The protein interactome of activated bivalent genes revealed the increase of the connectivity of unicellulars and (early) multicellulars, while circadian regulators were depressed. The mutual polyploidy-*c-MYC*-bivalent genes-associated protein network was organized by gene-hubs engaged in both embryonic development and metastatic cancer including driver (proto)-oncogenes of viral origin. Our data suggest that, in cancer, the atavistic shift goes hand-in-hand with polyploidy and is driven by epigenetic mechanisms impinging on development-related bivalent genes.

## 1. Introduction

Whole-genome duplications (WGD) and recurrent polyploidization, providing a source for gene divergence and adaptability to environmental changes, are central in the evolution of biodiversity [1,2,3]. Polyploid cells are also present in the tissues of vertebrates: in humans and other mammalians, cells with multiplied genomes appear during normal organogenesis of definitive and provisional organs (placenta, heart, brain, liver, skin, blood) [4,5,6,7,8,9,10]. Polyploidy also arises in response to stress, wounding, and in cancer [11,12,13,14,15,16,17,18].

The relationship between polyploidy and stemness, both found as typical features of aggressive tumours [19,20,21,22], may be associated with the re-activation of evolutionarily ancient programs. The observations show that malignant cells often acquire the phenotypes and reproductive behaviour of unicellular organisms through transient polyploidy “life-cycle”, which is reciprocally linked with a cell cycle [23,24,25,26,27]. This behaviour is characteristic for cancer cells resistant to therapeutic treatments [28].

The atavistic theory of oncogenesis suggests that cancer is a reversal from a multicellular to a unicellular state [7,29,30,31,32,33]. The two giant clusters were revealed in the human coexpression gene network (also based on bulk tissue analysis): a widely expressed cluster enriched in genes of unicellular origin enriching the cancer tissues and the other giant cluster of multicellular genes [34,35]. Phylostratigraphic tracking of the genes involved in cancer [36] suggests their link to the emergence of multicellularity in metazoan, many cancer genes have a viral origin and even the typical somatic mutations of the cancer genes can be traced as ancestral [37].

In turn, the studies of Trigos and colleagues [38,39] also showed that the genes of unicellular origin are overexpressed in human cancers as compared to their normal counterpart tissues, whereas the genes appearing at multicellular stages of evolution were downregulated. Moreover, in tumours, the interaction between the unicllularity and multicellularity gene networks is weakened [38,40].

The upregulation of the unicellular giant cluster in cancer cells was shown in the single-cells transcriptomes of various cancer types and in invasive as compared with non-invasive cancer [35,41]. These two clusters can be defined as “echoing” a proper dynamical attractor given, strictly speaking, they are not mutually exclusive (both in cancer and normal cell genes from both clusters are activated). The prevalence of ancient unicellular state of cancer cells with respect to normal tissue corresponds to a partial “going back” to the more ancient unicellular pattern preceding the rise of multicellularity. This implies we do not expect that cancer cells become ’identical to unicellular organism’ but that lose some phenotypic characters essential for the existence of an organized tissue like contact inhibition, the crucial role played by shape changes in cancer development is consistent with this view [42,43].

The epigenetic mechanisms favouring transition to unicellularity and cancerogenesis and, thus, the proposed evolution reversal of human cancer cells remain unclear. Since oncogenesis is frequently associated with polyploidy [2,26,44,45,46,47], we suggested the involvement of polyploidy in this process and addressed here the phylostratigraphy and protein networks of the genes differentially expressed in polyploid versus diploid tissues.

We choose this approach, because mammalian homologous tissues differ by cell ploidy levels [1,2,5,8,12]. Some species have predominantly polyploid heart and diploid liver (pig and primates), whereas others possess mainly polyploid liver and predominantly diploid heart (rodents) [1,5,8]. Here, we rely on this fact to generate a balanced factorial design with a criss-cross distribution of ploidy/tissue across human and mouse that allows to separate ploidy effect from both tissue and species influences (see Methods).

To promote understanding of the epigenetic nature of ploidy-associated gene regulation, we also investigated how polyploidy influences the expression of bivalent genes. Bivalent genes are characterized by epigenetic ambiguity, bearing in their promoters or enhancers two opposite epigenetic modifications of the histone H3, the repressing H3K27me3 and the activating H3K4me3, poised for transcription but capable for its rapid activation. These poised genes were established in embryonic stem cells [48,49] and are the main players in the early and post-implantation development and cell fate change. Changes of bivalent chromatin coincide with the increased expression of developmental genes in cancer and are also likely involved there in the epigenetic changes [50,51,52,53,54,55]. On a more general ground the notion of cancer as ‘development gone awry’ supports the focus on such genes [56].

The obtained results indicate that polyploidy activates unicellular metabolic pathways and ancient programs of development related to carcinogenesis. This evolutionary retour is accompanied by the activation of bivalent genes and deregulation of circadian rhythms. Altogether, these results provide evidence that polyploidy is an important driver of epigenetic changes linked to cancer that may reorganize gene regulatory networks.

## 2. Results

### 2.1. Polyploidy Causes Transition of Gene Phylostratic Balance towards Evolutionary Old Unicellular Phylostrata

To find out whether polyploidy affects the expression of genes of various evolutionary ages, we first applied pairwise cross-species transcriptome comparison to polyploid vs. diploid organs in human and mouse (human heart vs. mouse heart and mouse liver vs. human liver). Then we evaluated the effects of polyploidy at various thresholds of the expression difference. This evaluation gave 584 upregulated and 711 downregulated genes at two-fold difference and 1028 upregulated and 887 downregulated genes at a 1.3-fold difference (Tables S1 and S2).

**Figure 1:**
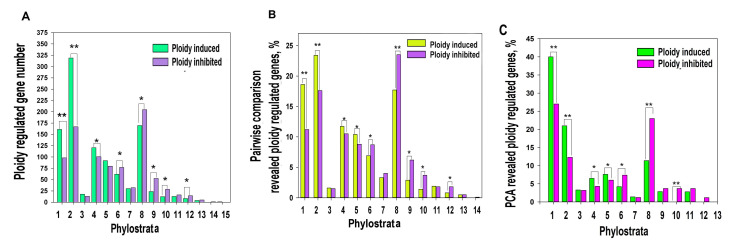
Ploidy associated gene distribution across evolutionary phylostrata. Phylostratigraphy of ploidy associated genes revealed by pairwise cross-species (criss-cross) comparison (**A**,**B**) and by PCA (**C**). This figure illustrates that polyploidy shifts the gene age balance of expressed genes from metazoan phylostrata (6–16, Bilateria and later) towards unicellular phylostrata (1–3) via transitional early metazoan phylostrata (4–5) * *p* < 0.05 for the difference, ** *p* < 0.01 for the difference. Gene expression difference above two-fold. The phylostrata are as follows: 1—cellular organisms, 2—Eukaryota, 3—Opisthokonta, 4—Metazoa, 5—Eumetazoa, 6—Bilateria, 7—Chordata, 8—Euteleostomi, 9—Amniota, 10—Mammalia, 11—Ttheria, 12—Eutheria, 13—Euarchontoglires, 14—Catarrhini, 15—Homininae.

Phylostratic distribution of ploidy-associated genes was analysed by grouping genes according to their age of evolutionary origin taken from [38]. Our data indicated that polyploidy is associated with the upregulation of ancient genes originating in unicellular ancestors (1-3 phylostrata) and the downregulation of genes starting with phylostratum 6 (Bilateria) and onwards, while the early multicellularity strata 4–5 (Metazoa and Eumetazoa) did not reveal a clear difference (Figure 1A,B).

To verify this result with a well-known traditional approach, we applied principal component analysis (PCA). PCA identified 103 upregulated and 96 downregulated genes having above two standard deviations. Of these genes, 78 and 87 genes demonstrated a more than two-fold expression difference (Tables S1 and S2). With excluding the tissue-specific component, the phylostratic distribution of PCA-revealed genes also indicated the activation by polyploidy of evolutionarily conserved programs (genes from phylostrata 1–3) and the suppression of young programs maintaining multicellularity (phylostrata 6–13, Figure 1C). The phylostrata 4–5 were up-regulated.

### 2.2. Polyploidy Activates Recapitulation of Evolutionary Developmental Programs Associated with Carcinogenesis

To investigate the functional consequences of ploidy-associated phylostrata rearrangement at the gene module level, we performed gene module enrichment analysis for genes with more than two-fold expression difference between polyploid vs. diploid organs. The results are presented in Table S3. For unicellular phylostrata (1–3) the main effects of polyploidy were the induction of modules related to drug ABC pump and drug metabolism (hsa02010;GO:0017144), protein and ribosome synthesis (hsa01230;hsa03010), oxidation-reduction (GO:0055114) and energy metabolism (both aerobic respiration and carbohydrate metabolism (GO:0019752; GO:0008152; GO:0044262; hsa00051). This picture is seen from the higher significance of enrichment and the larger gene number for upregulated genes than for downregulated ones. For the up-regulated by polyploidy early metazoan phylostrata (4–5) our data reveal the upregulation of modules related to embryonic development (GO:0009790), stem cell commitment (GO:0045165), pluripotency (hsa04550) and Kegg pathways of carcinogenesis (hsa05200; hsa05205). The phylostrata 6–8 were down-regulated by polyploidy. They are involved in the development of the multicellular organism complexity. The downregulated genes of the 6–8 strata were mostly enriched in biological processes related to immunity (GO:0002682), inflammation (GO:0050727) and communication (GO:0010646) that are the specific biological features of multi-cellularity. Consistently, multicellular phylostrata (10–15, i.e., Mammalia and later) were not enriched for gene modules depending on polyploidy.

The results of functional enrichment of PCA-ploidy-revealed genes for old phylostrata were consistent with the results of pair-wise cross-species comparison (Table S4). Thus, the carcinogenesis pathways favoured by polyploidy appear in the early metazoan (strata 4–5) in one pack with embryogenesis and cell fate change (stemness commitment), associated with asexual reproduction.

Similar changes in the general phylostratigraphic landscape shifted towards unicellularity found here for polyploidy were previously shown for tumorigenesis as such [38]. The authors also paid attention to the enrichment of the intermediate between unicellularity and multicellularity phylostrata with cancer driver genes [40]. In comparison with our data, this similarity suggests that polyploidy may favour the epigenetic evolutionary shift of normal cells to cancer. The interactome of *c-MYC* proto-oncogene provides a useful link to verify this suggestion because *c-MYC* in an important regulator of both ploidy and cancer.

### 2.3. c-MYC Induction Drives Polyploidy-Associated Transcriptomic Changes towards Unicellularity

*c-MYC* is a regulator of the normal cell cycle. However, when over-expressed it disjoins replication from cell division and favours polyploidy [57,58]. Moreover, while the overexpressed non-mutant (often amplified) *c-MYC* is a powerful reprogramming factor and oncogene, its downregulation in transgenic mice causes tumour regression [59,60,61,62].

The pleiotropic *c-MYC* is capable to drive the opposite processes, proliferation versus apoptosis and is acting as a master activator of the bivalent genes involved in the epigenetic regulation of development [60,63,64,65].

**Figure 2:**
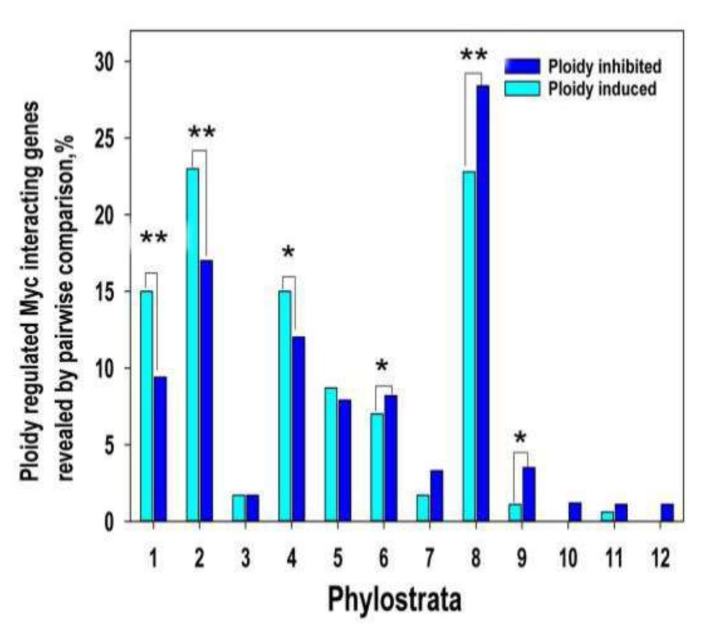
Ploidy associated *c-MYC* interacting gene distribution across evolutionary phylostrata. This figure illustrates that *c-MYC* -interacting genes repeat pattern of all ploidy related gene age distribution. Gene age is shifted from young multicellular phylostrata (6–16) towards unicellular phylostrata (1–3 phylostrata) via transitional metazoan phylostrata (4–5). * *p* < 0.05 for the difference, ** *p* < 0.01. Gene expression difference is above two-fold.

Here we evaluated the phylostratic distribution of ploidy-dependent *c-MYC* -interacting genes at two-fold expression difference (Tables S5 and S6) and explored its link through polyploidy with bivalent genes (Figure 2). The data on ploidy -Myc-ploidy-related gene phylostratigraphic changes repeat the ploidy-dependent relationships (compare with Figure 1)

It is clear that the non-mutant *c-MYC* should elaborate these effects in the epigenetic modus. Therefore, we paid attention to the fact that *c-MYC* controls gene transcription by activating bivalent genes.

### 2.4. The Phylostratigraphic Polyploidy-Associated Effect of c-MYC Is Associated with the Regulation of Bivalent Genes

**Figure 3:**
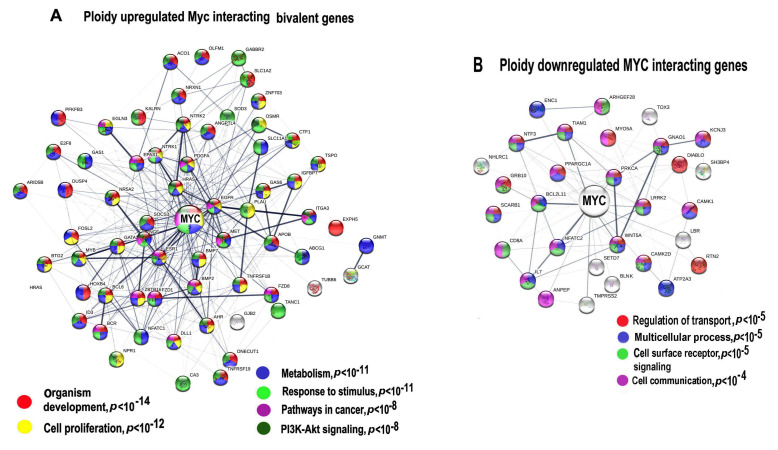
Protein interaction networks (PPIs) for ploidy associated *c-MYC* -interacting bivalent genes. PPI for upregulated (**A**) and downregulated (**B**) genes. Gene expression difference is above two-fold. The networks are constructed with server String. Gene pathway enrichment was found using the same server.

The genome-wide studies indicate that the overexpressed *c-MYC* demethylates the H3K27me3 repressive domain of bivalent genes and thereby switches these genes to the active state [63,64], it also activates Polymerase II paused in bivalent genes [53]. To find out whether overexpressed Myc also increases expression of ploidy-related bivalent genes, we first identified these genes among ploidy-regulated *c-MYC* interacting genes with expression difference above two-fold. Genes of interest were identified using the list of bivalent genes in human embryonic stem cells [54]. Our data identified 60 bivalent genes of 161 ploidy-upregulated Myc interactants and 27 bivalent genes among 101 ploidy-downregulated *c-MYC* interactants (Figure 3A,B, Tables S7 and S8).

Protein interaction network presented in Figure 3A indicates the Myc interacting and ploidy upregulated bivalent genes. Gene module functional enrichment analysis indicated that these genes are enriched in pathways of development, proliferation, stress response, and pathways in cancer with high significance. Figure 3B that presents *c-MYC* interacting ploidy downregulated genes shows that the cell surface-mediated functions (transport, reception, communication) are downregulated.

It is important to note that the network for the upregulated bivalent genes shows higher connectivity (13.5 connection per a node) compared to the downregulated genes (only 5.13 connections per a node), which implies that *c-MYC* and polyploidy- induced activity of bivalent genes considerably increases epigenetic plasticity of protein interaction network. It should be noted that the overexpressed functional pack again included a tandem of development and carcinogenesis modules, which we have found in the ploidy-related gene phylostratigraphic analysis. Therefore, we decided to extend the investigation of bivalent genes in relation to ploidy and gene phylostrata through the entire transcriptome.

### 2.5. Ploidy-Associated Genes Are Enriched in Bivalent Genes of the Entire Genome, Prevailing among Unicellular and Eumetazoan Phylostrata.

We compared the genes with above two-fold expression difference dependent on polyploidy (vs. diploidy) with bivalent genes in human embryonic stem cells [54]. Among the 584 up and 711 downregulated by ploidy genes (Tables S1 and S2) we found 267 (45.7%) and 222 (31.6%) of up-and down-regulated bivalent genes, respectively (Tables S9 and S10). The results of the binomial test indicate that bivalent genes enrich ploidy-upregulated genes compared to the entire transcriptome (3024 vs. 14093) with high significance (*p* < 10^−16^ binomial test). For the downregulated genes the significance was *p* < 10^−8^ (for 21.3 vs. 31.2%), respectively. These results indicate that polyploidy mostly induced bivalent genes.

**Figure 4:**
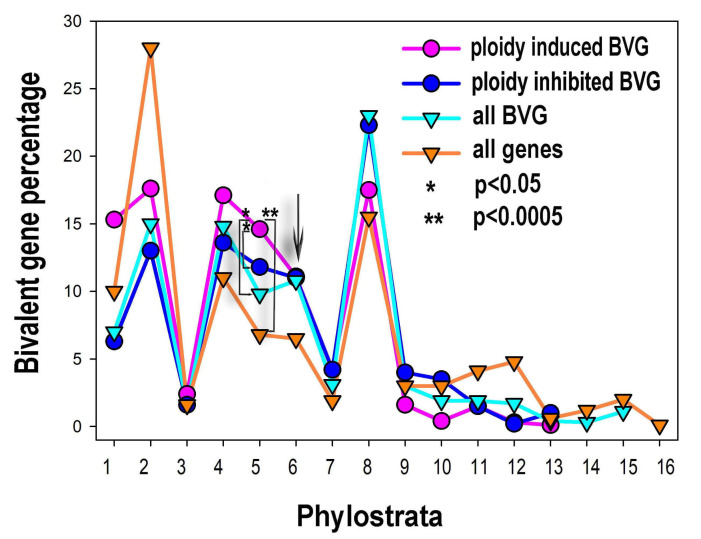
The percentual proportions of gene origins and distribution of bivalent genes (BVG) in the phylostratic tree of life (strata 1–16) and the effect of polyploidy on it. The upregulation of bi-valent genes by polyploidy includes strata 1, 2 (unicellularians), stratum 4 (metazoa) and, prominently, stratum 5 (eumetazoa—the appearance of embryo, germ layer, and gastrulation). The arrowed cross-point starting down-regulation of bivalent genes by polyploidy in late metazoa falls upon stratum 6 (bilateria).

The percentage of phylostratigraphic distribution of ploidy associated bivalent and common genes is shown in Figure 4. The bivalent genes, comprising ~21% of the human genome, originated in evolution in all phylostrata but in the larger proportion with development of multicellularity, from phylostratum 4 (Metazoa) to 8 (Euteleostomi). The distribution of the expression of the ploidy upregulated bivalent genes by phylostrata is different from that for all bivalent genes. It is clearly seen that these genes repeat the general effect of all ploidy-regulated genes and *c-MYC*-ploidy regulated genes. Among the ploidy upregulated bivalent genes prevail the proportions of phylostrata 1 (Prokaryota) to 5 (Eumetazoa). Proportions of phylostrata 6 and 7 (Bilateria and Chordata) are ambivalent. Proportions of phylostrata 8 (Euteleostomi) to 10 (Mammalia) show clear decrease (Figure 4). The ploidy downregulated bivalent genes generally repeat the pattern for all bivalent genes.

### 2.6. Protein Interaction Networks for Ploidy-Associated Bivalent Genes Are Involved in the Upregulation of the Developmental and Carcinogenesis Genes and the Downregulation of the Networks Related to Differentiation Biological Quality and Circadian Clock

**Figure 5:**
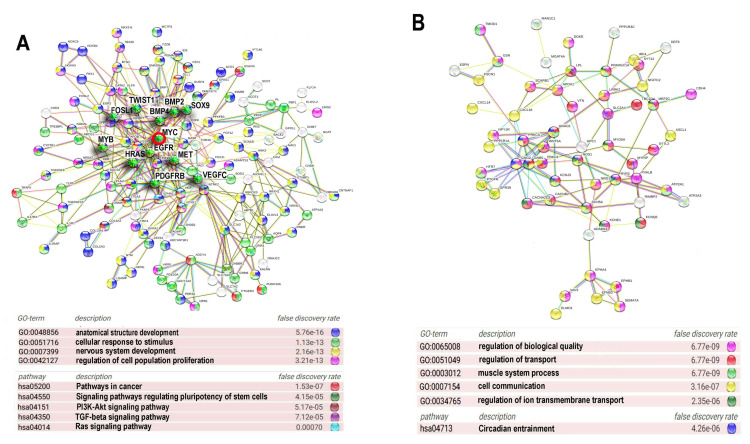
Protein interaction networks for ploidy associated bivalent genes. The most connected components of protein interaction networks of upregulated (**A**) and downregulated (**B**) bivalent genes constructed using STRING server. Gene expression difference is above two-fold. Gene module analysis and node degree evaluation was done using the same server. The titles of GO biological processes are given with for false discovery rate. The important drivers of carcinogenesis are marked with green asterisks. Myc oncogene is marked with a red circle.

We constructed protein interaction networks (PPIs) for ploidy up- and downregulated bivalent genes marked with H3K27me3 and H3K4me3 in human ESC using the String server. We also included *c-MYC* that is not a bivalent gene, but it is of particular interest because this important oncogene is an inducer of bivalent genes [64]. The networks were constructed for genes with above two-fold expression difference. Then we extracted the whole connected component from the up- and downregulated networks and clustered them using the same server. For the upregulated genes the network contains 165 bivalent of 267 ploidy upregulated bivalent genes (62%). For the ploidy downregulated bivalent genes it contains 63 genes of 222 (24%). As in the case of *c-MYC*, the striking difference in gene numbers of the connected components of up- and downregulated networks (163 vs. 63) by bivalent genes in the whole transcriptome, despite approximately the same numbers of up- and downregulated genes, shows that the upregulated by ploidy bivalent genes create essentially more functional connections and master regulators compared to the downregulated genes. Below we provide a functional description of the up and downregulated PPIs (Figure 5A,B). It is clearly seen that the upregulated network contains more hub regulators than the downregulated network (29 vs. 13).

The PPI for the ploidy upregulated bivalent genes (Figure 5A) is enriched in the GO biological processes related to the development of anatomical structures (GO:0048856) and the nervous system (GO:007399), proliferation (GO:0042127), response to stress and stimulus (GO:0051716), and in KEGG pathways involved in the regulation of pluripotency (hsa04550), pathway of carcinogenesis (hsa05200) and several pro-carcinogenic and development related pathways, including *PI3K-AKT* (hsa04151) *TGFB* (hsa04350) and *RAS* (hsa04014) signalling pathways (Figure 5A). It is important to note that this PIN unifies proteins involved both in development and metastatic cancer that comprise dense functional nucleus of the induced network and are the hub proteins with high degree of connectivity originating from 1–6 phylostrata genes (Table 1). The PPI for the downregulated genes is enriched for GO biological processes participating in transport (GO:0051049, GO:0034765), cell communication (GO:0007154), regulation of biological quality (GO:0065008), muscle system processes (GO:003012) and circadian entrainment (hsa04713). Thus, the functional picture provided by PPI for the upregulated sgenes indicates that ploidy-related bivalent genes are associated with the induction of embryonic developmental programs including drivers of carcinogenesis (Figure 5A, Table 1). PPI for the downregulated genes illustrates the depression of circadian clocks.

### 2.7. The Relation of Cancer Driver Genes to Polyploidy, Bivalency and Their Phylostratigraphic Origin

**Figure 6:**
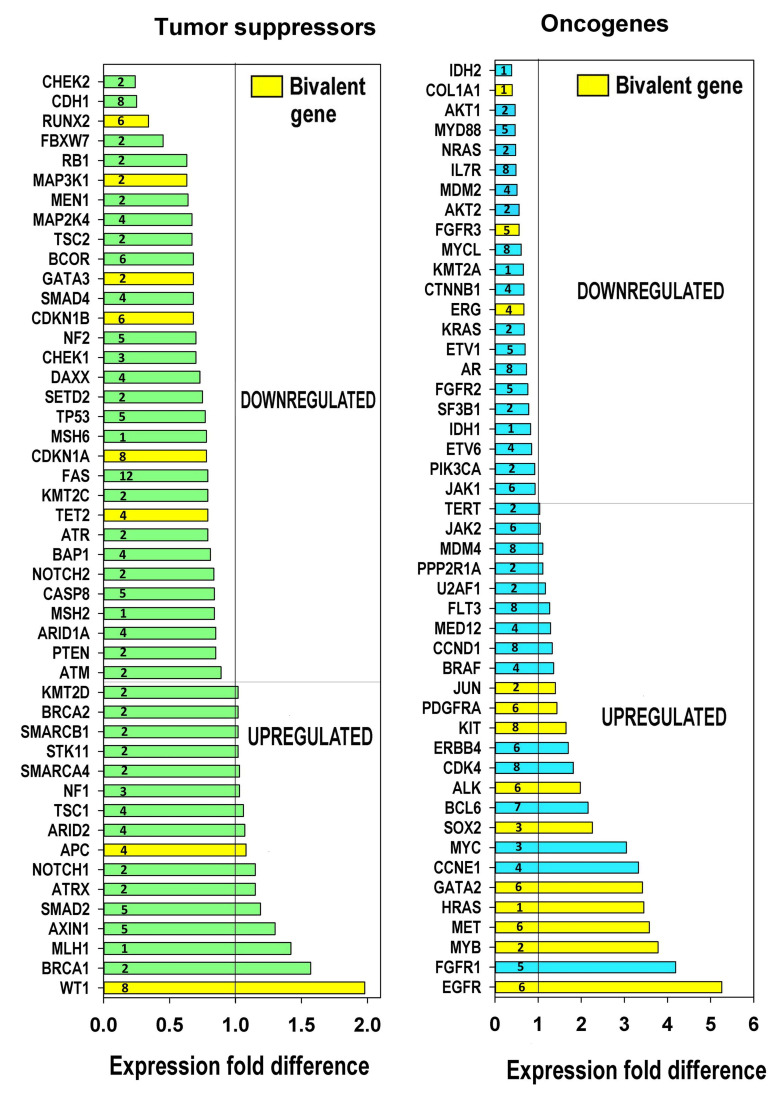
Ploidy associated tumor suppressors and oncogenes. This figure illustrates the downregulation of tumour suppressors, the upregulation of oncogenes and the enrichment of the induced oncogenes with bivalent genes *p* < 0.05, binomial test. The phylostrata numbers of gene evolutionary origins are indicated.

To obtain more evidence concerning features of carcinogenesis, we evaluated the expression of tumour suppressors and oncogenes in relation to polyploidy based on the list from [78]. The obtained data presented in Figure 6, together with phylostratic origin of genes, indicates that polyploidy is associated with preferential downregulation of tumour suppressors and strong upregulation of many oncogenes. It is important to note that the upregulated oncogenes were more strongly enriched with bivalent genes than the downregulated oncogenes and tumour suppressors (*p* < 0.02 for all comparisons, binomial test). The notably down-regulated by polyploidy tumour suppressors belong to DNA damage response: *ATR*, (2nd phylostratum), *ATR* (2nd), *CHEK2* (2nd), *CHEK1* (3rd), *TP53* (5th)—however, its protein inhibitor *MDM2* (seen in the column for oncogenes) is also inhibited; apoptosis executor Fas, prominent tumour suppressor *PTEN* (2nd) and the E-cadherin-associated gene *CDH1* (8th phylostratum). Thus, cell communication is suppressed by polyploidy, while cells become more tolerant to DNA damage and inhibit apoptosis.

Among the prominent oncogenes upregulated by polyploidy, we find a master transcription factor (TF) of early stress response Jun (2nd phylostratum), *KIT*-tyrosine-protein-kinase (6th); among 3–5-fold upregulated oncogenes, we see *GATA* 2 TF (6th) responsible for embryonic development, *c-MYC* TF (3rd, viral origin); *MYB* TF (2nd, viral origin); *MET* Tyrosin-protein-kinase (6th)— often a target for avian leukemia retrovirus [79] *HA-RAS* G-protein (1st phylostratum, viral origin)—the most common oncogene in at least 30% of all tumours and, finally, its upstream regulator *EGFR* (6th); both are bivalent (Table S9).

Clearly, in this analysis, we have used the transcriptome data from polyploid tissues, where the activated protooncogenes were not mutated. In addition to their relation to bivalency and embryonic development, we see that the main tumour suppressor *TP53* and strong oncogene *EGFR* belong to the intermediate phylostrata 5–6, while many important driver oncogenes of this list activated by polyploidy are from the earliest evolutionary phylostrata. Furthermore, the most often involved in carcinogenesis prominent complementary pair of oncogenes *c-MYC* and *HRAS* (phylostrata 1–3) are of the most ancient origin.

## 3. Discussion

Despite extensive development of therapies, metastatic cancer disease remains incurable, resulting in high morbidity and mortality [80]. The association of aggressive incurable cancer with polyploid giant cells has been shown [28]. The extraordinary resistance to extinction therapies suggests that cancers recapitulate the phylogenetic endurance acquired in the long evolution of the lifeforms on Earth [81]. The cancer genome sequencing projects compromised, to some extent, a somatic mutation theory [82,83]. In recent times, the epigenetic aspects of the whole genome regulation in cancer stepped forward and are now intensively explored. One of these aspects is associated with typical for cancers whole genome duplications, or polyploidy (and aneuploidy inevitably linked to it), as a feature characteristically accompanying tumour growth and aggravating with tumour aggression [18]. The genetic sequences of polyploidy and aneuploidy in the microevolution of cancer were in the focus of studies for rather a long time [84,85]. However, the epigenetic aspects of aneuploidy became also attractive [86,87]. Considering a possible atavistic effect of cancer polyploidy, the phylostratigraphic analysis considering the evolutionary origin of genes and the change of their expression balance may be helpful.

Therefore, an interesting epigenetic approach may represent the study of the phylostratigraphic effect of polyploidy of not mutant normal mammalian tissues. In this paper we have investigated these issues by bioinformatical means using transcriptome data of polyploid versus diploid cells of normal human heart and mouse liver.

We have revealed that polyploidy changes the phylostratigraphic balance of the mammalian cellular network in favour of the enhanced expression of the evolutionary most ancient gene phylostrata—prokaryotic, early eukaryotic and early metazoan (1–5 phylostrata) up to bilateria, after which the trend is changed to the opposite. Although found here in normal polyploid tissues, this shift is surprisingly similar to the changes revealed for tumours when compared with normal counterparts [34,35,36,38]. With this revelation we arrive to the immediate conclusion: this epigenetic shift in tumours to the unicellular and early multicellular forms of life is associated or at least highly favoured by polyploidization.

The question arises—how it is regulated (or rather disregulated)? One of the mechanisms found in our study—it is by use of so-called bivalent genes, the epigenetically modified by histone H3 repressive and activating modifications of the same gene promoter or enhancer, namely by their prevailing activation. The particularity of bivalent genes comprising ~21% of human genes and employed for cell fate change in development is that they are poised (paused) but can be rapidly activated. That means that they favour cell fate change by the rules of non-equilibrium thermodynamics, in particular by critical state transition [88].

It is known now that the chromatin conformation at the supranucleosome and higher levels of the genome architecture is much a subject of the biophysical processes which assemble the active and inactive chromatin in two separate self-organizing high-order domains [89,90,91,92,93]. Self-organization is an important driving force of evolution [94]. On one hand, polyploidy is well known as driving gene diversification and speciation in evolution [1], which is a slow process. On the other hand, its epigenetic effect causing rapid reorganization of the transcriptional regulatory network after genome duplication was shown [95,96].

It can be reasoned that multiplication in polyploid cells of the identical chromosome alleles with the identical epigenetic status of bivalent genes should not just linearly increase their epigenetic effect proportionally to gene dosage but potentiate it by the thermodynamics of structural self-organization (phase transition). The role of the closely-juxtaposed DNA fibres’ electrostatic forces yielding fine-tuned structure-specific recognition and pairing [97,98] should also contribute in the chromatin self-organization by polyploidy. A similar thought on the potentiating structural effect of additional DNA by the chromatin modifiers and transcription factors has been proposed earlier [99,100].

The Kegg “pathways in carcinogenesis”, along with the genes known as being mutants in cancers [101], also include many developmental genes. Here we revealed that this developmental component is largely introduced through the polyploidy-dependent enrichment of the expression networks with the activated bivalent genes upregulated by *c-MYC* master hub (stratum 3). While in general gene evolution the proportion of bivalent genes increases from phylostrata 3 to 8, the polyploidy is mostly affected by enrichment with expression of bivalency in the phylostrata of Prokaryote and Eumetazoa (1—5). Very likely, that it is achieved by Myc up-regulating both polyploidy and bivalency. Thus, the carcinogenic *H-RAS-c-MYC* feed-back loop (strata 1–3) linked to developmental genes of early metazoans (strata 4–5) enriched with developmental pathways becomes involved, while stratum 6 (Bilateria) becomes a cross-point of the ambivalence (with repulsion from vertebrates).

*c-MYC–mtH-RAS* complementary pair has been distinguished in the early cancer chemical carcinogenesis research in a two-hit theory of cancer with Myc determining immortality and cancer initiation and *H*-*RAS* mutant in the codon 12 or 61 accomplishing cancer promotion [71,102]. Afterwards, ample studies have extended this theory by demonstrating that a potent oncogenicity of Myc can be further enhanced by collaborations with not only *RAS* mutant but also many extracellular growth stimuli that activate *RAS*, such as epidermal growth factor and its receptor (*EGFR*) or transforming growth factors [103,104]. The found phylostratic effect of ploidy-associated *c-MYC* collaborating with bivalent genes, e.g., both *H*-*RAS* (stratum 1) and *EGFR* (stratum 6) may explain its critical role in cancer initiation when overexpressed, and cancer regression when locked [59,60,62]. This phylostratic effect induced by the oncogenic potency of *c-MYC* linked to induced polyploidy, metabolic stemness, and bivalency likely creates the evolutionary “cancer attractor” postulated by Kauffman [94] as situated close to the summit of metaphoric Waddington Hill of the development potential [105,106]. Moreover, the upregulated *c-MYC* cooperates with mutant ras through programming inflammation and suppressing the tumour immunity [61], and *c-MYC* acts by cell selection promoting proliferation versus apoptosis depending on the supply of growth factors [60]. Likely, this trigger can occur through asymmetric cell division of epigenetically diverged bi-nuclear cells [107,108]. In fact, the main actors of this complex “story” including the complementary *c-MYC* and *RAS* pair can be found in Table 1 describing tumour and stromal components (like angiogenesis) of the polyploidy-dependent “cancer attractor”.

Interestingly, cnidaria (Hydra), an organism where the basic processes of the multicellularity were established represents phylogenetically the oldest organisms, where the tumours were revealed [109].

The early eukaryotic gene cluster in human genome is more stable than the cluster of complex metazoans (starting from vertebrates and peaking in acquired number of new genes at stratum 8) [38,40,41]. The unicellular gene network is responsible for basic cellular functions and also for resistance to extinction elaborated through series of earth catastrophes in billion years—we highlighted here that the genes responsible for DNA damage response, drug resistance, early stress response, and the proto-oncogenes of the viral origin, are from the 1–3 phylostrata. These likely act through feedback loops as the driving belts epigenetically attracting the cells from the basin of early metazoans into a unicellularity network.

On the other hand, the basic evolutionary processes of the early embryonal development of mammals are conserved from their origin in Eumetazoa, stratum 5 [110]. Therefore, aggressive cancer associated with polyploidy and multinuclearity often bears the features of the early embryo [111,112], including such inalienable components of morphogenesis as cell motility and angiogenesis. Moreover, through polyploidy, the *TP53* cancer mutants can acquire the phenotypical features of more ancient unicellular eukaryotes (“amoeboidisation”) [24,27,33,108,113,114], in line with the atavistic theory of cancer [29,30,33,115,116], confirmed by gene phylostratigraphic analysis [38] and here.

The early metazoans are tolerant to polyploidy because use it reversibly in their life-cycles [117]; it is sufficient to mention *Amoeba vulgaris* and *Entamoeba histolytica* [33,118,119] and amoebal slime molds [120]. When associated with polyploidy, as we found, the bivalent genes increase the connectivity and basin of attraction and become embedded in the network with the oncogene hubs of the viral origin, such as *c-MYC*, *H-RAS*, MYB—originated and having strong established interactions in the older strata (1–2–3). Moreover, polyploidy permits DNA replication but stops cell division and induces suppression of the bivalent genes regulating circadian rhythms, which entrain many cellular processes, including cell cycles [121]. The cell-autonomous circadian timers established already in cyanobacteria are composed of a transcription–translation-based auto-regulatory feedback loops [122]. Apparently, this detrainment is an important factor causing along with epigenetic bibivalency a metastable dynamic state, which can also favour a reshape and glide from the multi-cellular to unicellular gene expression network.

However, de-polyploidisation, in turn, can probably cause the back-shift of the transcription network: the two cycles, polyploidy (life-cycle-like) and diploidy (cell cycle) are reciprocally linked in tumours [23,87]; the recovery diploid fraction reciprocally downregulates the expression of amoebal genes in polyploidized human breast cancer treated with anticancer drugs; in addition, the amoebal giant cells can play a “nursing” function for the reproductive cell line [27]. Without any doubt, the constitutively activating mutations of oncogenes increase, push and likely stabilize the epigenetic shift to unicellularity. Such prominent tumour suppressors as *TP53* and *HYPPO/YAP* pathway, which also serve as guardians of the barrier to polyploidy [123], normally prevent the carcinogenic epigenetic shift to unicellularity caused by overcoming this barrier.

## 4. Methods

### 4.1. Data Sources and Comparative Criss-Cross Analysis

In this study we applied the method of reciprocal pair-wise cross-species transcriptome comparison that we used previously [12,57,124]. We choose this approach, because mammalian homologous tissues differ by cell ploidy levels [1,2,5,8,12]. Certain species have predominantly polyploid heart and diploid liver (pig and primates), whereas others possess mainly polyploid liver and predominantly diploid heart (rodents) [1,5,8]. Humans have highly polyploid hearts where cardiomyocytes contain nuclei with 4–16 genomes, whereas mouse hearts, on the contrary, consist of cardiomyocytes with mostly diploid nuclei [12]. At the same time, human hepatocytes are mostly diploid, whereas mouse hepatocytes contain nuclei with 4–8 genomes [5,12]. This inverse pattern of polyploidization enables identifying pure ploidy specific effects since it removes species-specific and tissue-specific noise [12]. In this study we applied pairwise cross species transcriptome comparison and principal component analysis (PCA) to comparisons of polyploid vs. diploid tissues: human heart vs. mouse heart and mouse liver vs. human liver. In the present work there are three main advancements over previous studies. Firstly, here we made the functional analysis of all ploidy-associated genes based on the whole-transcriptome data. Previously, the analysis was limited to the *c-MYC* interactome only [58,124]. Secondly, we studied the gene evolutionary origin (phylostratic distribution of all polyploidy-associated genes), which is important for cancer research in the context of the main contradiction arising between the unicellular and multicellular level features in tissue organization [34,38,41]. Thirdly, we addressed possible epigenetic mechanisms underlying ploidy-related changes, focusing on the bivalent gene expression and interactome. The work builds upon the idea of reciprocal cross-species comparison. The cross-species approach is informative because evolutionary distance enhances the contrast allowing the polyploidy-specific signature to appear [125].

The signal-to-noise filtration, where signal corresponds to polyploidy and noise to species- and tissue-specific signatures, is especially important for investigation of polyploidy because polyploidy preserves gene-dosage balance and thus may exert only weak and idiosyncratic effects on gene expression [126]. We performed the reciprocal pair-wise cross-species comparison using the transcriptomic data for polyploid vs. diploid organs. Specifically, we compared human heart (polyploid) vs. mouse heart (diploid) and mouse liver (polyploid) vs. human liver (diploid). The data were taken from the database obtained using RNA-seq [127].

The processed data were taken from the Supplement at the journal site [127]. We matched human-mouse orthologous genes and normalized the data in pairwise comparisons (human-mouse heart, human-mouse liver) using “quantile” normalization implemented in the “limma” package [128], as was done previously [124,129]. Finally, there were 13,913 orthologous gene pairs for which we found stratigraphic data in Trigos et al. [38]. The transcriptomic data were uniformly obtained for all tissues and species [127]. This database was developed specially for interspecies comparisons (in particular, the samples were taken from the same parts of organs).

We detected the genes whose expression was changed in the same direction with regard to ploidy both in the heart and liver. In these comparisons the genes should have higher (or lower) expression in both polyploid tissues (human heart and mouse liver) compared with corresponding diploid tissues (mouse heart and human liver). Since we compared two different tissues in opposite directions in different species (human vs. mouse in the case of heart, and mouse vs. human in the case of liver), the tissue-specific and species-specific effects were presumably removed.

The one-to-one human-mouse orthologous genes were obtained from the NCBI database [130]. The expression levels of orthologous genes were analysed using the “limma” package specially developed for revealing differentially expressed genes in whole-transcriptome analyses [128]. A comparison of different software packages showed that limma is the method of choice for goals similar to those pursued in our work [131]. The data were normalized with “quantile” normalization implemented in “limma”. The differential gene expression (with statistical significance) was determined using the “voom” limma procedure. Then, we selected the genes with different expression contrasts between polyploid and diploid tissues as indicated in Results.

### 4.2. Principal Component Analysis

To determine whether the results of cross-species comparison can be confirmed by another approach, we applied principal component analysis (PCA) to the raw data matrix having samples as variables and genes as statistical units. The idea is to confirm the gene-by-gene a priori approach with a data-driven strategy, letting a ploidy-specific principal component to emerge from the data. The principal components are orthogonal to each other by construction; the data-driven emergence of a “ploidy” component distinct from tissue and “species” components is equivalent to an unsupervised normalization for tissue and species effects [132]. The genes endowed with extreme scores on such a “ploidy” component are the “image in light” of tissue and species independent ploidy effect on transcription pattern.

For this propose we used the same transcriptomic data for human and mouse heart and liver. This approach enabled us to evaluate the impact of shared variation (driven by housekeeping genes), species-specific, tissue-specific and ploidy-specific variables as separated mutually independent components [133].

As a result, we obtained two lists of genes demonstrating statistically significant ploidy-associated variation (above two standard deviations) for human and mouse heart and liver. Then these gene lists were subject to gene module enrichment analysis and the modules that are regulated by ploidy were identified. PCA is a purely geometrical non probabilistic procedure [132]. This means that each gene is simply projected into a rotated space (in this case having the same dimension of the original one, thus, with no loss of information) spanned by mutually orthogonal axes (components) extracted in decreasing order of explained variance. This unsupervised purely geometrical approach gave rise to a loading pattern mirroring the batch, tissue, species, ploidy a priori classification so demonstrating the tenability of the existence of “ploidy specific” genes. At this point the identification of genes having a score > |2| as markers of the ploidy component corresponds to the usual 95% confidence intervals (component scores are equivalent to z scores). The actual *p*-values for phylostratigraphic analysis were based on relative enrichment of genes pertaining to different stratigraphic level based on the entire gene ontology. The samples having same tissue and species origin are practically coincident [see 132] for the presence of a common gene expression ideal profile for same tissue independent samples). RNA-seq analysis was based on the shared gene products across different samples; these shared genes were the pivots for coupling the relative vectors.

The species-specific and tissue-specific effects were automatically removed by PCA [124]. As a matter of fact the principal component analysis applied in an unsupervised way to the gene expression profiles, generated a four-component solution correspondent to batch effect (PC1, 45.6% of variance explained), tissue-effect (PC2, 30.5% of variance); species-specific effect (PC3, 15% of variance) and ploidy effect (PC4, 9% of variance). We concentrated on genes having relevant score (higher than |2| corresponding to two standard deviations from mean) on PC4. Principal components are each other independent by construction, therefore batch, tissue and species confounding are eliminated by the spectral decomposition of the dataset.

### 4.3. Analysis of Gene Modules

To determine which biological modules were over-represented among the ploidy-associated genes, we applied a double control. We tested the genes from all three datasets with higher and lower expression in polyploid vs. diploid tissues for enrichment of Gene Ontology (GO) categories and molecular pathways with regard to all human-mouse orthologous genes. The enriched GO categories and molecular pathways were found using the hypergeometric distribution of probability (implemented in R package) as previously [134,135]. GO categories were taken from GO database [136]. For each GO category, we collected all its subcategories using Gene Ontology acyclic directed graphs, and a gene was regarded as belonging to a given category if it was mapped to any of its subcategories. As a source of molecular pathways, the NCBI BioSystems was used, which is a most complete compendium of molecular pathways from different databases [130]. Redundancy of this compendium was removed by uniting entries with identical gene sets. The adjustment for multiple comparisons was done according to the method by [137]. This procedure gives a q-value, which can be considered a *p*-value corrected for multiple tests.

### 4.4. Protein-Protein Interaction Network

The protein-protein interaction networks (PPI) were constructed and visualized using the STRING server [138]. The analysis of network connectivity and the identification of causal regulators in modular and network organization were done with the same server.

### 4.5. Phylostratigraphic Analysis of Ploidy Associated Genes

The attribution of genes to phylostrata was taken from [38]. The phylostrata are as follows: 1—cellular organisms, 2—Eukaryota, 3—Opisthokonta, 4—Metazoa, 5—Eumetazoa, 6—Bilateria, 7—Chordata, 8—Euteleostomi, 9—Amniota, 10—Mammalia, 11—Theria, 12—Eutheria, 13—Euarchontoglires, 14—Catarrhini, 15—Homininae, 16—*Homo sapiens*.

### 4.6. Identification of Ploidy Associated Bivalent Gene

To find out how polyploidy affects bivalent gene expression landscape, we evaluated the expression of ploidy-associated genes that are known to be marked by bivalent chromatin in stem cells. The list of these genes was taken from [54].

## 5. Conclusions

The obtained results indicate that polyploidy activates the unicellular pathways of resistance to extinction fusing with ancient programs of ontogenesis related to carcinogenesis which originated in the early metazoa while suppressing the expression of genes from the late metazoa. This evolutionary retour is favoured by the activation of bivalent genes and deregulation of circadian rhythms. The data highlight the paramount role of polyploidy in the atavistic origin of cancer, the reason for the incurability of metastatic cancer, and indicate to targeting polyploidy for overcoming resistance to therapies.

## Figures and Tables

**Table 1:** Characteristics of the central hubs of Myc-related network of proteins encoded by bivalent genes upregulated by polyploidy (presented in Figure 5A).

Gene/ Protein	Folds Up-Regulation	Number of Connections	Phylo Stratum	Function Involvement in Cancer	References
*BMP4*	3.24	26	4	Development, cell motility, oncogene	[66]
*BMP2*	3.84	14	5	Development, cell motility, oncogene	[67]
*c-MYC*	3.2	29	3	Stemness, proliferation, apoptosis, polyploidy induction, driver oncogene	[68]
*MET*	3.7	15	6	Embryonic development, proliferation; when mutationally activated is involved in multiple cancers	[69]
*MYB*	3.8	8	2	Development of colon epithelial progenitors, high in gastric, colon, and breast cancer	[70]
*HRAS*	3.6	19	1	Proliferation, senescence, germ development, driver oncogene	[71,72]
*EGFR*	4.94	34	6	Epithelium development, driver oncogene	[73]
*PDGFRB*	2.23	15	6	Development, cancer-related angiogenesis	[74]
*VEGFC* (*C*)	2.88	12	5	Blood vessels development, cancer-related angiogenesis	[74]
*SOX9*	3.86	15	4	Germ (male) and skeleton development	[75]
*TWIST1*	2.53	11	2	Mesoderm development, motility, metastatic cancer	[76]
*FOSL1*	2.19	11	5	Early stress response, reinforces Myc, oncogenesis	[77]

## Supplementary Materials

Supplementary materials can be found at https://www.mdpi.com/1422-0067/21/22/8759/s1.
